# Impact of a structured multicomponent educational intervention program on metabolic control of patients with type 2 diabetes

**DOI:** 10.1186/s12902-017-0222-2

**Published:** 2017-12-15

**Authors:** Maria do Rosário Pinto, Pedro Miguel Dinis Santos Parreira, Marta Lima Basto, Lisete dos Santos Mendes Mónico

**Affiliations:** 1 Higher School of Health, Polytechnic Institute of Santarém, Santarém, Portugal; 2 Higher School of Nursing of Coimbra, Santarém, Portugal; 3 Higher School of Nursing of Lisbon, Santarém, Portugal; 4 University of Coimbra, Psychology and Sciences of Education Faculty, Santarém, Portugal

**Keywords:** Diabetes Mellitus, Complex Interventions, Health Outcomes

## Abstract

**Background:**

Diabetes is one of the most common metabolic disorders, with a high prevalence of patients with poor metabolic control. Worldwide, evidence highlights the importance of developing and implementing educational interventions that can reduce this burden. The main objective of this study was to analyse the impact of a lifestyle centred intervention on glycaemic control of poorly controlled type 2 diabetic patients, followed in a Community Care Centre.

**Methods:**

A type 2 experimental design was conducted over 6 months, including 122 adults with HbA1c ≥ 7.5%, randomly allocated into Experimental group (EG) or Control Group (CG). EG patients attended a specific Educational Program while CG patients frequented usual care. Personal and health characterization variables, clinical metrics and self-care activities were measured before and after the implementation of the intervention. Analysis was done by comparing gains between groups (CG vs EG) through differential calculations (post minus pre-test results) and Longitudinal analysis.

**Results:**

Statistical differences were obtained between groups for HbA1c and BMI: EG had a decrease in 11% more (effect-size r2 = .11) than CG for HbA1c (*p* < .001) and 4% more (effect-size r2 = .04) in BMI (*p* < .05). When controlling for socioeconomic characteristics and comorbidities that showed to be associated to each parameter in pre-test, from pre to post-test only EG participants significantly decreased HbA1c [Wilks’ ʎ = .702; F(1,57) = 24.16; *p* < .001; ηp2 = .298; observed power = .998]; BMI values [Wilks’ ʎ = .900; F(1,59) = 6.57; *p* = .013; ηp2 = .100; observed power = .713]; systolic Blood pressure [Wilks’ ʎ = .735; F(1,61) = 21.94; *p* < .001; ηp2 = .265; observed power = .996] and diastolic Blood pressure [Wilks’ ʎ = .795; F(1,59) = 15.20; p < .001; ηp2 = .205; observed power = .970].

**Conclusions:**

The impact of a structured multicomponent educational intervention program by itself, beyond standard educational approach alone, supported in a Longitudinal analysis that controlled variables statistically associated with clinical metrics in pre-test measures, has demonstrated its effectiveness in improving HbA1c, BMI and Blood pressure values.

**Trial registration:**

RBR-8ns8pb. (Retrospectively registered: October 30,2017).

## Background

Diabetes, one of the most common metabolic disorders, is a public health concern rising with a widespread significance all over the world, with a clear tendency to increase in the next decades [1–4].

In Portugal, the country where this study was conducted, the estimated prevalence of diabetes is 13.1%, mainly in the age group between 20 and 79 years, mostly in type 2 sort. According to the National Diabetes Observatory, from the diabetic people with records of glycosylated haemoglobin (HbA_1c_) in Nacional Health Service (NHS), 21.8% have values over 8% [5]. This demonstrates that, despite the considerable research and health organizations and educators’ investment in this area, poor metabolic control is a major problem that seems to remain in a significant percentage of Portuguese patients.

Poor metabolic control has proven to be linked to the development of multiple complications, both acute and chronic, whose probability of appearance and aggravation can be reduced by improving glycaemic control and disease management [6].

Contributing to the prevention of earlier mortality and the decrease of patients’ quality of life [7] management of patients with complex diseases as type 2 diabetes, is a cornerstone of delaying and preventing complications and an important step towards improving clinical and metabolic outcomes, appropriate utilization of health care resources and the resulting health gains.

The Portuguese NHS responded to this imperative need by defining and implementing the concept of Therapeutic Education, an educational process, prepared, developed and carried out by trained health professionals, in order to enable the patient and their family to deal with a chronic illness situation, such as diabetes and with the prevention of its complications, aiming to maintain patients’ quality of life in a synergetic combination with additional therapeutics effect [8].

In addition, evidence has highlighted the importance of developing and implementing interventions that can reduce this burden, by using intensive strategies to control blood glucose levels [9, 10], pointing lifestyle correction as one of the most important aims for people with type 2 diabetes [4, 11, 12].

Described as a key-step for metabolic control in all the algorithms for diabetes treatment, educating for an adequate lifestyle is, currently, a recommendation present in all guidelines, due to the growing evidence of its effectiveness [13–15].

However, although this has been widely explored, as international literature reveals, little evidence has been produced about educational intervention effectiveness concerning the Portuguese reality. The interest and importance of evaluating the impact of educational intervention among poorly controlled type 2 diabetic patients lead to this research, structured according to the guidance on the development, evaluation and implementation of complex intervention to improve health, upon Medical Research Council framework [16, 17], aiming to analyse the impact of a lifestyle centred intervention on glycaemic control of poorly controlled type 2 diabetic patients, followed in a community care centre.

Guided and structured on the principles of Therapeutic Education, described on Table 1, an Educational Program was designed throughout a prior study, supported on theoretical and evidence based previous knowledge, along with an insightful analysis of the environment care reality [17].

**Table 1:** Therapeutic Education in Diabetes [8]

Grounds	• The right to education of the diabetic patient about their illness and the ways of controlling their situation • Patient co-responsibility within disease management /Patient empowerment • Importance of enabling the diabetic and their family to daily related disease decision-making, making them as independent as possible from health services and professionals, who are expected to progressively, play a consultant role. • NHS facilities rational use, in order to make the joint efforts of health professionals profitable.
Educational process and Evaluation	Educational process is understood as the methods, or means by which resources are used to achieve educational objectives, involving several components which must be documented, in order to be subject to evaluation: • individual evaluation, taking into account the unique characteristics that allow the individualization of educational needs • establishment of short, medium and long-term objectives; • development of an education plan and its implementation; • evaluation and follow-up Evaluation of the educational process is fundamental and must be made in relation to the knowledge and abilities acquired by patients, to health professionals as trainees, and to the strategies, objectives and outcomes applied
Professionals involved	Doctors, nurses, psychologists, dietitians/nutritionists, pharmacists and other technicians.
Expected competencies of the professionals	• adapt professional behaviour to the patients and their illness, either individually or in groups • adapt professional role to that of the other care teams with whom they cooperate • communicate empathically with patients • recognize objective and subjective needs of patients • take into account the patients’ emotional state, previous experiences and representations • educate the diabetic in the management of treatment, crises, interfering factors and in the adequate use of available health, social and economic resources • to support the diabetic in their learning and in the adaptation of their lifestyle • to select patient education tools, integrating them into the learning process • take into account the educational, psychological and social dimensions of continuing education • to evaluate therapeutic effects of education in clinical, biological, psychological, educational, social and economic dimensions, making adjustments whenever necessary • to periodically evaluate and improve professional’s performance in this area
Patients’ expected competencies	• select self-care objectives • modify diet/ food habits • take the medication prescribed appropriately • adjust physical activity
The programmes	These programs must comply with process and results quality criteria and should be designed according to the different types of health professionals involved in the Educational process.

**Fig. 1:**
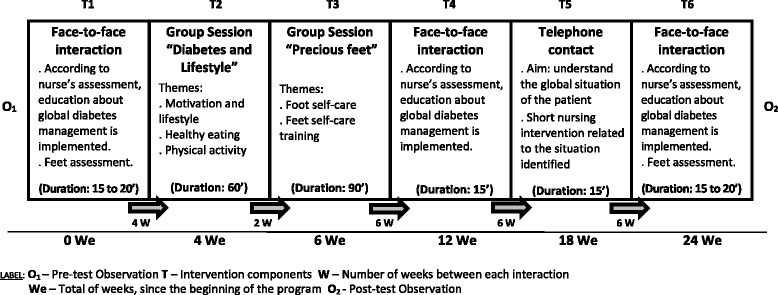
Educational Program implemented

This type of intervention has to be focussed on diabetes self-management education, a process in which knowledge and skills are provided for patients to perform self-care on a daily basis, as optimizing glycaemic control though self-management is the keystone of care in diabetic patients [18]. Thus, having present that the various components of an educational intervention necessary to embrace the several dimensions of self-management peculiarities [19], we designed a complex intervention that includes individual interaction between diabetes educators and patients; group approach, a main theme throughout the activity which was theoretically structured and planned as a trigger to motivate pair discussion, and telephone intervention, organized in a sequential global program [20, 21], schematically described on Fig. 1.

After a piloting procedure [16, 17], the Program was implemented in a Community Care Centre, offered to a group of poorly controlled type 2 diabetic patients [Experimental Group (EG)], while another group continued to receive the usual intervention, used in this health care institution, corresponding to the moments identified in the figure above as T_1_, T_4_ and T_6_ [Control Group (CG)]. By analysing the outcomes of this procedure, new contributions regarding the effects of a structured educational intervention program on metabolic control among Portuguese poorly controlled people with type 2 diabetes are highlighted. More specifically, we intend to ascertain whether a multicomponent educational program focused on lifestyle management strategies: (a) is significantly associated with metabolic control; (b) is effective on improving metabolic control; and (c) whether metabolic control is maintained after controlling for socioeconomic and health individual variables (comorbidities).

## Methods

### Participants

The sample is composed of two groups of adult/middle age patients with type 2 poorly controlled diabetes (HbA_1c_ ≥ 7.5%/58.5 mmol/mol), followed in a Community Health Centre of the Portuguese NHS, using age and HbA_1c_ as relevant control selection variables.

**Fig. 2:**
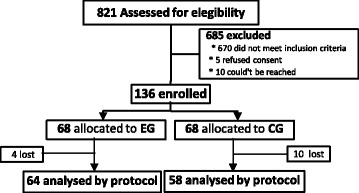
Flow diagram of the recruitment process

All the patients who fulfilled the relevant control criteria (*n =* 151) were invited during a routine consultation to participate in our study. From those, 136 agreed to do so, and were randomly allocated by the researcher into EG or CG in series of 2 patients for each group (2 for EG/ 2 for CG, and so on). The researcher was the only one in possession of the process number of the patient, without no further information (see flow diagram on Fig. 2).

The sample calculation was performed using the sample size determination formula based on the estimated proportion of the population with known p (proportion of the population of individuals belonging to the category we are interested in studying; *p* = 13.1% in 2014) and q (proportion of the population of individuals not belonging to the category we are interested in studying, 1 – *p* = 86.9%) [22]. So with a confidence level of 90% and α = 0.05, we would need at least 123 (rounded up from the figure of 123.22) subjects (our sample was composed of 136 participants).

Of the 136 participants, 122 concluded all the phases of the procedure (mortality rate of 10.3%): 64 in EG, between 35 and 67 year-old [*M* = 58.95 years, *SD* = 7.22 years] with HbA_1c_ between 7.5% (58.5 mmol/mol) and 14% (129.5 mmol/mol) [*M* = 9.07%; *SD* = 1.47%] and 58 in CG, between 35 and 67 year-old [*M* = 55.97 years; *SD* = 8.28 years]; with HbA_1c_ between 7.5% (58.5 mmol/mol) and 12.4% (112 mmol/mol) [*M* = 8.5%; *SD* = 1.04%].

Over 6 months, EG participants attended the Educational Program described on Fig. 1, while patients from CG frequented the usual care developed in the Community Care Unit (only 3 face-to-face moments, corresponding to moments T_1_, T_4,_ and T_6_ identified in the same figure).

### Materials

We collected clinical metrics [HbA_1c_, body mass index (BMI) and blood pressure (BP)], apart from personal and health characterization variables. Self-care activities were assessed using the Summary of Diabetes Self-care Activities Scale [23, 24] that was translated and validated for Portuguese patients [25], a measure that has proven to be sensible to self-care behavioural change [24].

### Variables

As independent variable we considered the Group (CG versus EG), as EG followed the educational program procedures and CG followed the usual intervention developed in Community Centre. Dependent variables were HbA_1c_, BMI and BP measures. As control variables, supported on evidence based knowledge previously produced, we used socioeconomic characterization and personal health variables, namely, academic qualifications, cohabitation, economic difficulties, labour occupation, comorbidities (hypertension and other cardiovascular diseases, depression and anxiety, over weight and obesity, diabetes late complications and other comorbidities), adherence to diabetes self-care activities (*SDSCA* score) and to its dimensions (General diet, Specific diet, Exercise, Blood-glucose testing, Medications and Foot care).

### Ethics and procedures

After Ethics Committee for Health from Regional Health Administration of Lisbon and Tagus Valley approval, the trial was registered with the ID 039/CES/INV/2014, following the Portuguese legislation. All the consort guidelines for reporting a clinical trial were followed, including informed consent, obtained from all patients.

Data were collected in two moments. The first time before the beginning of the educational program (pre-test) and the second after educational intervention program was finished (post-test). Anthropometric and clinical data were collected from clinical records and *SDSCA* was self-administered (an average of approximately 20 min of filling time). For all analyses we considered a probability of type I error (α) = 0.05.

### Data analysis

This study has a type 2 experimental design [26], involving the measures of dependent variables before and after the implementation of an intervention and the manipulation of an independent variable (educational program designed vs. usual intervention).

Data were processed in IBM SPSS 22.0. For each dependent variable, the skewness and kurtosis didn’t show values indicating violations of the Normal Distribution (|Sk| < 3 and |Ku| < 8) [27, 28]. The Levene’s Test for Equality of Variances showed variance homogeneity across groups (*p* > .05).

## Results

### Clinical metrics within EG and CG in pre and post test

The comparison between pre and post-test CG and EG showed a significant decrease in all clinical metrics’ measures only in EG (see Table 2). For CG the only significant decrease was for diastolic blood pressure (BPd).

**Table 2:** Clinical metrics results for CG and EG, in pre and post-test: Paired-samples *t-test*

	CG (*n* = 58)				*t*(57)	EG (*n* = 64)				*t*(63)
	Pre-test		Post-test			Pre-test		Post-test		
	*M*	*SD*	*M*	*SD*		*M*	*SD*	*M*	*SD*	
HbA1c (%/mmol/mol)	8.53/69.7	1.04	8.48/69.2	1.08	.63	9.08/75.7	1.47	8.29/67.1	1.26	4.74**
BMI	30.69	5.42	30.73	5.71	−.28	30.70	5.98	30.20	5.84	2.60*
BPs	144.22	16.22	141.10	13.10	1.64	146.16	15.05	139.25	13.06	4.53**
BPd	82.53	9.80	79.98	8.38	2.18*	81.66	10.24	77.66	7.92	3.81**

*HbA1c* Glycosylate Haemoglobin, *BMI* Body Mass Index, *BPs* Systolic Blood Pressure, *BPd* Diastolic Blood Pressure, *M* Mean, *SD* Standard deviation, *t* t-test

* *p* < .05; ** *p* < .001

After these first results, we took the differential calculations (post minus pre-test results) of HbA_1c_, BMI, BPs and BPd measures as dependent variables, in order to compare the intervention gains between groups (CG vs EG), controlling for clinical metric results in pre-test. An independent-samples *t*-test was performed, taking as independent variable the group (CG versus EG) and as dependent variables the differential results (see Table 3). Statistical differences were obtained between groups for HbA_1c_ and BMI: EG had an estimated decrease in 11% (effect-size *r* ^2^ = .111) more than CG for HbA_1c_ and above 4% in BMI (effect-size *r* ^2^ = .038).

**Table 3:** Clinical metrics differential results for CG and EG: independent-samples *t-test*s and effect sizes

	CG		EG		*t*(120)	*Cohen’s*	*Effect-size*
	*M*	*SD*	*M*	*SD*		*d*	*r*
HbA1c (%)	−0.05	0.65	−0.79	1.33	3.93**	.707	.333
BMI	0.04	1.16	−0.50	1.54	2.18*	.396	.194
BPs	−3.12	14.53	−6.91	12.19	1.56	.283	.140
BPd	−2.55	8.93	−.3.80	7.98	0.81	.148	.073

*M* Mean, *SD* Standard deviation

*p < .05; **p < .001

Despite these results, as our study groups (EG and CG) had significant differences on HbA_1c_ levels at baseline, and literature points out several variables that can interfere in this process, we decided to conduct a Longitudinal analysis, an approach which permitted the examination of the effect of the Educational Program on clinical metrics from pre-test to post-test, while controlling for variables associated with these metrics. The differential calculations of dependent variables were used in order to control the differences found in the pre-test.

### Socioeconomic characteristics and comorbidities associated with clinical metrics

As literature identifies, some variables are associated with clinical metrics in people with diabetes. In Table 4, correlations between these variables, diabetes control for significant associations are presented for control and experimental groups in pre-test. The variables which presented a significant association with clinical metrics were taken as control variables in the longitudinal analysis.

**Table 4:** Intercorrelations between socioeconomic characteristics/comorbidities and clinical metrics, on pre-test

	HbA1c	BMI	BP systolic	BP diastolic
Cohabitationª	.030	.031	−.062	−.051
Academic qualificationsª	−.048	.196*	−.090	.179^*^
Economic difficultiesª	.238^**^	.033	.119	.047
Labour occupationª	.060	−.064	−.271^**^	−.025
Hypertensionª	−.072	.099	.002	−.167
Cardiovascular diseasesª	−.067	.045	.091	.081
Depressionª	.046	.224^*^	.082	−.057
Anxietyª	.030	.064	.042	.121
Diabetes complicationsª	.041	−.025	.101	−.127
Obesityª	.076	.429^**^	.050	.179^*^
Overweightª	−.227^*^	−.191^*^	−.118	−.099
Other pathologiesª	.077	−.054	−.040	−.168
Self-care adherence score	−.320^**^	−.016	−.159	−.213^*^
General diet	−.385^**^	.017	−.237^**^	−.195^*^
Specific diet	−.213^*^	−.025	−.104	−.126
Exercise	−.143	.006	−.125	−.171
Blood-glucose testing	.073	−.088	.007	−.085
Foot care	−.171	.066	.011	−.077
Medications	−.179^*^	−.059	−.092	−.033

ª dummy variable; * *p* < .05; ** *p* <. 001

### Clinical metrics from pre to post-test in CG and EG: Longitudinal analysis controlling for socioeconomic characteristics and comorbidities

Considering a longitudinal analysis, we began by examining the effects of educational program on clinical metrics from pre-test to post-test, while controlling for socio economic characteristics and comorbidities of participants which showed to be associated with these parameters in pre-test. We considered group (CG with usual intervention vs. EG with educational program) as a between-subjects factor, clinical metrics as within-subjects variables (from pre to post-test), and socioeconomic characteristics and comorbidities as covariates.

We performed four mixed ANCOVAs, one for each dependent variable (HbA_1c,_ BMI, BPs, and BPd). According to intercorrelations found, for HbA_1c_ we controlled for economic difficulties, overweight, self-care adherence score, general and specific diet and medication. For BMI we controlled for academic qualifications, depression, and for associated diagnosed diseases (overweight and obesity). For BPs we controlled for occupational labour and general diet and for BPd for academic qualifications, obesity, self-care adherence score and general diet.

**Fig. 3:**
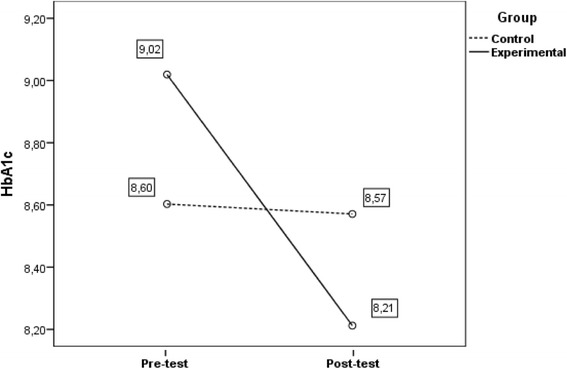
Evolution of HbA_1c_ from pre to post-test as a function of educational program. Evolution of HbA_1c_ from pre to post-test as a function of educational program (CG vs. EG) when controlling for socioeconomic characteristics and comorbidities. Covariates appearing in the model are evaluated at the following values: Economic difficulties (dummy) = .34; Overweight (dummy) = .20; Self-care adherence score = 3.8438; General diet = 3.28; Specific diet = 4.16; Medication = 6.34

For haemoglobin (HbA_1c)_, despite the statistical removal of the effect of the covariates, we found a significant interaction between within-subjects variables and the between-subjects factor, Wilks’ *ʎ* = .893; *F*(1114) = 13.63; *p* < .001; *η*_*p*_^*2*^ = .107; observed power = .955 (see Fig. 3). Through usual intervention done in the Community care Unit, CG didn’t decrease their HbA_1c_ values from pre to post-test, Wilks’ *ʎ* = .993; *F*(1,51) = 0.36; *p* = .550; η_p_^2^ = .007; observed power = .091. Inversely, we noted that EG dropped significantly their HbA_1c_ from pre to post-test, Wilks’ ʎ = .702; *F*(1,57) = 24.16; *p* < .001; *η*_*p*_^*2*^ = .298; observed power = .998. We concluded that after controlling for these covariates, only experimental group participants significantly decreased HbA_1c_, with and effect size of 30%.

**Fig. 4:**
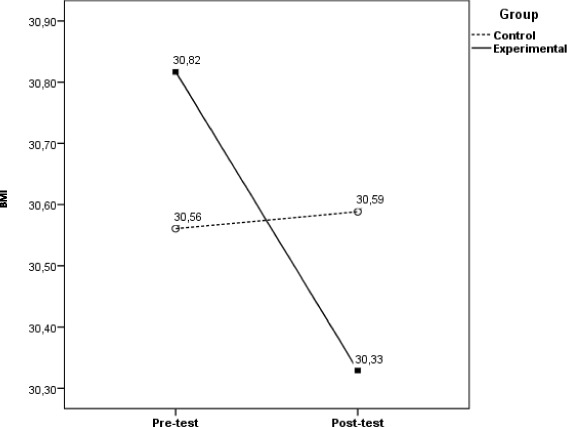
Evolution of BMI from pre to post-test as a function of educational program. Evolution of BMI from pre to post-test as a function of educational program (CG vs. EG) when controlling for personal characteristics and comorbidities. Covariates appearing in the model are evaluated at the following values: Labour occupation (dummy) = .73; General diet = 3.28

When BMI was taken as dependent variable, and we controlled covariates associated with it, we also found a significant interaction between within-subjects variables and the between-subjects factor, Wilks’ *ʎ* = .967; *F*(1116) = 4.01; *p* = .048; *η*_*p*_^*2*^ = .033; observed power = .510 (see Fig. 4). Control group didn’t decrease their BMI values from pre to post-test, Wilks’ *ʎ* = .999; *F*(1,53) = 0.08; *p* = .782; *η*_*p*_^*2*^ = .001; observed power = .059. In reverse, experimental group dropped significantly their BMI from pre to post-test, although with a less effect size, Wilks’ *ʎ* = .900; *F*(1,59) = 6.57; *p* = .013; *η*_*p*_^*2*^ = .100; observed power = .713.

As for BP as dependent variables, when we control covariates associated with diastolic and systolic blood pressure, the interactions between within-subjects variables and the between-subjects factors were significant, due to a decreased in both groups from pre to post-test, [BPs _within-subjects variables_: Wilks’ *ʎ* = .908; *F*(1118) = 12.03; *p* = .001; *η*_*p*_^*2* =^ .092; observed power = .930; BPd _within-subjects variables_: Wilks’ *ʎ* = .920; *F*(1116) = 10.05; *p* = .002; *η*_*p*_^*2* =^ .080; observed power = .882].

**Fig. 5:**
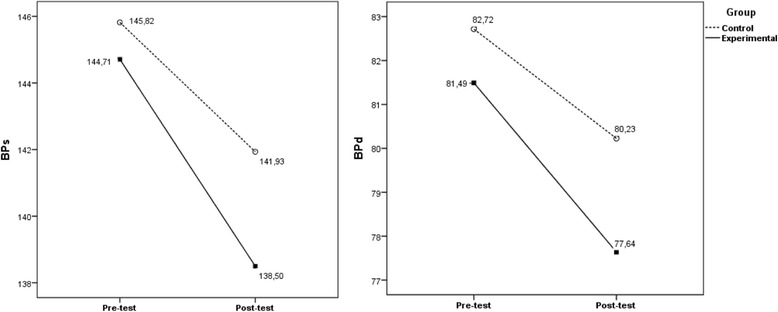
Evolution of BPs and BPd from pre to post-test as a function of educational program. Evolution of BPs and BPd from pre to post-test as a function of educational program (CG vs. EG) when controlling for socioeconomic characteristics and comorbidities. Covariates appearing in the model are evaluated at the following values: BPs: Overweight (dummy) = .20; academic qualifications (dummy) = .54; depression (dummy) = .16; obesity (dummy) = .37; BPd: academic qualifications (dummy) = .54; obesity (dummy) = .37; self-care adherence score = 3.8438; general diet = 3.28

Once more, this decrease was only statistically significant for EG with effect sizes over 21% [BPs _within-subjects variables:_ Wilks’ *ʎ* = .735; *F*(1,61) = 21.94; *p* < .001; *η*_*p*_^*2*^ = .265; observed power = .996; BPd _within-subjects variables:_ Wilks’ *ʎ* = .795; *F*(1,59) = 15.20; *p* < .001; *η*_*p*_^*2*^ = .205; observed power = .970] as for CG we obtained: BPs _within-subjects variables:_ Wilks’ *ʎ* = .954; *F*(1,55) = 2.67; *p* = .108; *η*_*p*_^*2*^ = .046; observed power = .362 and BPd _within-subjects variables_: Wilks’ *ʎ* = .918; *F*(1,53) = 4.72; *p* = .34; *η*_*p*_^*2*^ = .082; observed power = 569 (see Fig. 5).

## Discussion

This study, an experimental controlled research, was conducted with the purpose of determining whether an educational specific program was effective in improving metabolic control of a group of poor controlled type 2 diabetic patients, as evidence has shown that metabolic control can be accomplished by multicomponent interventions based on Therapeutic Education, addressing healthy lifestyle and self-management [18, 29–35].

Although published evidence-based exists, this is a research area little explored in Portuguese reality with few available studies centred on educational intervention effectiveness assessment. Portugal is one of the European countries where often health expected outcomes fail to meet guidelines due to multiple known barriers to health professionals’ intervention and patients’ outcomes achievement [18, 36–38].

The findings of our study clearly support previous studies on improvement of HbA_1c_ levels through diabetes education [33, 39–41]. Throughout a first analysis it can be noted that, between pre and post-test, clinical metrics were sensible to the educational intervention. Emphasis is, however, that only in EG were identified statistically significant differences in these variations.

The most important metabolic control indicator for type 2 diabetic patients, glycosylated haemoglobin, is only significantly reduced in EG participants, with an estimated decrease of 11% more than in CG (differential calculations, controlling for Glycosylated haemoglobin in pre-test). The same applies to BMI variation, another cornerstone for diabetes health related outcomes, although this variation has a minor estimated decrease and its clinical value may be of lower significance. Blood pressure was the other clinical metric analysed in this study, due to the well-known relation between hypertension and diabetes, a condition that often affects people with type 2 diabetes. Results show both systolic and diastolic values have decreased from pre to post test, although without statistical significance.

Impact of the educational program by itself is supported in the Longitudinal analysis, when variables statistically associated with clinical metrics in pre-test measures were controlled. Notwithstanding the statistical removal of the effect of the covariates, a significant interaction between within-subjects variables and the between-subjects factor remains, leading to the conclusion that only in experimental group participants a significantly decreased in clinical metrics can be verified, as identified in other studies [42].

With distinct effect sizes, we confirmed experimental group participants reduced significantly clinical parameters from pre to post-test. EG participants registered a statistical significant decrease of HbA_1c_ values with an effect size of 11%; of BMI, although effect size is lower (3%) and of BP (systolic −27%, diastolic – 21%). CG participants didn’t have a significant decrease in HbA_1c_ values (*p* = .550), in BPs (*p* = .108) or in BPd (*p* = .34) and BMI increased from pre to post-test, although it didn’t have statistical significance (*p* = .782). These outcomes also are in line with the ones from other studies [33, 39–41].

Furthermore, our results sustain the impact of a structured multicomponent educational intervention on improving clinical metrics, beyond standard educational approach alone [40, 43–45], as all the participants received educational intervention, regardless of the group they were allocated to and results evidence differences between groups which are statically significant.

## Conclusions

It is known that often health expected outcomes fail to meet guidelines, patients’ expectations and needs, due to multiple known barriers to health professionals’ intervention and patients’ outcomes achievement^[^ [18, 36–38].

Education and lifestyle modification are identified as critical components for metabolic and clinical control in diabetic patients. Evidence-based reports have emphasized the importance of education for type 2 diabetic patients and educational programmes have confirmed their role in glycaemic control and decreasing diabetes associated complications [46].

The accuracy and innovative contribution of the present study is intended to be the assessment of the effectiveness of a specific educational program, designed upon an exploratory trial, where detailed educational needs of a group of Portuguese people with type 2 diabetes, were identified and taken into account in the definition of the Complex Intervention.

The multicomponent educational program, structured on the principle of Therapeutic Education, has proven its effectiveness, especially with regard to metabolic control, by lowering HbA_1c_ with statistical evidence between groups (CG vs EG), result reinforced when correlated variables were controlled.

Nevertheless, these results should be interpreted with some consideration due to the limitations of the study, mainly related to the baseline difference in HbA_1c_ levels between groups (EG and CG). There was even an aleatory patient distribution into groups, which leads us to suggest more research with Portuguese patients, analysing the impact of these programs in health gains, especially if randomization can be achieved.

## Abbreviations

ADA: American Diabetes Association
BMI: Body mass index
BP: Blood pressure
BPd: Diastolic blood pressure
BPs: Systolic blood pressure
CDA: Canadian Diabetes Association
CES: Comissão de Ética para a Saúde (Health Ethics Committee)
CG: Control Group
EG: Experimental Group
et al.: et alia (and others)
HbA1c: Glycosylated haemoglobin
IBM SPSS: International Business Machines- Software Package for Social Sciences
IDF: International Diabetes Federation
INV: Investigação (Research)
M: Mean
MRC: Medical Research Council
NHS: Nacional Health Service
SD: Standard deviation
*SDSCA*: Summary of Diabetes Self-Care Activities
